# The Impact of Structural and Functional Parameters in Glaucoma Patients on Patient-Reported Visual Functioning

**DOI:** 10.1371/journal.pone.0080757

**Published:** 2013-12-03

**Authors:** Christoph Hirneiß, Lukas Reznicek, Michaela Vogel, Konrad Pesudovs

**Affiliations:** 1 Ludwig-Maximilians University, Department of Ophthalmology, Klinikum der Universität Muenchen, Campus Innenstadt, Munich, Germany; 2 NH&MRC Centre for Clinical Eye Research, School of Medicine, Flinders University of South Australia, Adelaide, South Australia, Australia; Dalhousie University, Canada

## Abstract

**Background:**

To evaluate the impact of structural changes of the retinal nerve fiber layer (RNFL), and visual field loss, on functional impairment assessed by patient-reported visual functioning in glaucoma.

**Methods:**

Patients with glaucomatous optic nerve damage were enrolled in this cross-sectional study. Peripapillary RNFL thickness was obtained with spectral-domain optical coherence tomography (SD-OCT). Function was assessed by patient-reported visual functioning using the Rasch-calibrated Glaucoma Activity Limitation 9 (GAL-9) questionnaire and standard automated perimetry. The impact of peripapillary RNFL loss on functional impairment was analyzed with correlation and linear regression analyses.

**Results:**

A total of 176 eyes from 88 glaucoma patients were included. The SD-OCT assessed temporal-superior and temporal-inferior RNFL sector of the worse eye revealed significant correlation with the GAL-9 scores (r=-0.298, p=0.011 and r=-0.251, p=0.033, respectively). In mutivariate regression analysis, the best predictors for patient-reported visual functioning were visual acuity of the better eye and mean defect of the worse eye (R^2^=0.334), while structural parameters could not enhance the prediction of GAL-9 scores.

**Conclusions:**

Self-reported visual functioning of patients with glaucoma is better predicted by visual performance data than structural parameters. However, some structural changes of the worse eye are significantly correlated with patient-reported visual functioning.

## Introduction

Glaucoma is one of the leading causes of blindness with more than 70 million people affected worldwide[1]. However, blindness from glaucoma can be prevented by early diagnosis and treatment. Structural changes in glaucoma can be detected in the peripapillary retinal nerve fiber layer (RNFL), in the retinal ganglion cell layer complex and in the optic nerve head. Functional impairment of patients with glaucoma includes defects of the visual field with consecutively decreased visual functioning. Numerous studies have shown a correspondence between structure and function across the spectrum with all stages of glaucoma [2-4]. 

Precise measurements of the RNFL thickness are feasible with optical coherence tomography (OCT)[5], particularly with high resolution spectral domain (SD) OCT imaging. A relationship between RNFL thickness and functional parameters has been demonstrated[3]. [4] A number of studies have investigated the relationship between global perimetric indices (e.g. mean deviation, MD) and patient-reported visual functioning [6,7], but the relationship between structural changes in patients with glaucoma and patient-reported visual functioning is less clear. This relationship seems logical given the fact that structural changes affect vision, which in turn affects functioning; therefore, both measures, those of structure and vision, should be correlated with visual functioning. The nature of these correlations will depend upon the extent to which the measures of structure and the measures of vision capture deficits of importance for visual functioning. Glaucoma patients often report a diminished quality of life and visual functioning[8]. [9] In early glaucoma, quality of life is challenged by the need for continuous therapy, regular consultations and to some extent stressful repeated examinations (e.g. perimetry)[10,11]. In advanced glaucoma, patients´ quality of life is reduced due to visual field defects and the loss of functioning these cause[12]. Assessing the impact of the disease for the glaucoma patient is vital for optimizing individual advice and therapy. A number of patient-reported outcomes (PROs) exist for measuring quality of life or visual functioning in glaucoma[13-18]. The Glaucoma Activity Limitation 9 (GAL-9) is a well evaluated glaucoma-specific questionnaire designed to measure visual functioning[19]. [20,21] Importantly, that the GAL-9 is the only glaucoma-specific PRO to be successfully revalidated using Rasch analysis[19]. This is important for PRO scoring with the estimation of interval-scaled measures from ordinal data facilitating parametric statistical analyses such as relationship testing with linear regression[22]. 

This study was performed to evaluate the structure/function/patient-reported visual functioning relationships in patients with glaucoma. Structural peripapillary RNFL thickness was obtained using the SD-OCT, function was assessed with standard automated perimetry and visual acuity (VA), and patient-reported visual functioning was measured with the GAL-9. A secondary aim was to look for differences in the predictive value of structural and functional parameters depending on weather the better or the worse eye is regarded. 

## Patients and Methods

This was a cross-sectional investigation conducted in a German university-affiliated glaucoma center: the glaucoma unit of the Department of Ophthalmology, Ludwig-Maximilians-University, Munich, Germany. Potential study participants, patients with glaucoma attending for a regular review, were approached on a consecutive basis. All study participants underwent a full ophthalmic examination, including objective and subjective refraction, slit-lamp biomicroscopy, intraocular pressure (IOP) measurement with Goldmann applanation tonometry, gonioscopy, dilated fundus examination by indirect ophthalmoscopy and central corneal thickness (CCT) measurement. Inclusion criteria were age >18 years and a spherical refraction between -7.0 and +5.0 diopters. Patients with all stages of primary and secondary glaucomatous optic nerve damage undergoing all types of treatment or surgery were included. However, cases with secondary glaucoma associated with systemic disorders were excluded to minimize a potential confounding influence of comorbidity. Patients with a visual acuity of less than 20/50 in the better eye were excluded, as the items of the GAL-9 require at least minimal visual function for utilizable results. Further exclusion criteria included diabetes or history of other ocular or neurologic diseases. Approximately every tenth consecutive patient could not be enrolled due to unreliable standard automated perimetry or not meeting the 15 dB threshold for image quality in SD-OCT. 

Glaucomatous eyes were defined as those with reliable abnormal standard automated perimetry (at least 3 adjacent points had a >= 5 dB or at least 1 point had a >= 10 dB loss in compared numerical map) and optic nerve damage (rim thinning, notching, excavation, or retinal nerve fiber layer defect). Ethical approval of the study was obtained from the Institutional Review Board of the University Eye Hospital Munich in Germany and all the patients who agreed to participate signed a consent form. The study adheres to the tenets of the Declaration of Helsinki. 

### GAL-9 testing

There have been two recent reviews of PRO measures for glaucoma patients[23,24] that identified five superior quality disease-specific questionnaires. We considered each of these PROs and elected to use the Glaucoma Activity Limitation 9 (GAL-9), derived from the Glaucoma Quality of Life questionnaire (GQL-15) originally developed by Nelson et al.[20] This was because the GAL-9 focusses purely on visual functioning and contains a large number of; this appeared to offer the best prospects for correlating with structure metrics. In recent studies, the GAL-9 was analyzed in the German translation showing excellent measurement precision[19]. However, the items showed suboptimal targeting to the ability of the persons because, on average, they were too easy. Poor targeting is an inevitable problem for PRO questionnaires in glaucoma[25], including the widely used visual functioning questionnaire (VFQ-25)[26], because the overwhelming majority of patients with glaucoma have normal vision in at least one eye and excellent patient-reported visual functioning. Accepting this premise, the GAL-9 is the best available instrument for use in this study. The psychometric evaluation of the GAL-9 is explained in detail elsewhere[19]. All the items in the GAL-9 are scored on a 5-category difficulty scale ranging from 1 to 5 with labels of “no difficulty”, “a little bit of difficulty”, “some difficulty”, “quite a lot of difficulty” and “severe difficulty.” An additional category “Do not perform for non-visual reasons” was scored as missing data for the final analysis. The GAL-9 responses were transformed into person estimates using Rasch analysis. Rasch analysis is a probabilistic mathematical model which estimates item difficulty, person ability and the threshold for each response category on a single continuum logit scale. For this analysis, the person with higher ability (people less affected by the disease) and items of greater difficulty are located on the negative side of the logit scale and vice versa. In a prior analysis, it was shown that the GAL-9 enables unidimensional measurement of visual functioning with excellent psychometric attributes[19]. The use of Rasch analysis removes noise from the measurement and increases the likelihood of detecting significant relationships between variables[27]. In this study, the German version of the GAL-9 was self-administered by the patients in the clinic before the clinical examination or visual field testing began.

### SD-OCT testing

SD-OCT-based assessment of the RNFL provides a technological advance in the diagnosis of glaucoma[28]. The different SD-OCT instruments have similar abilities to detect glaucomatous RNFL damage[29]. For SD-OCT assessment of the RNFL, a Spectralis OCT was used (Spectralis HRA&OCT; software version 5.2.0.3, Heidelberg Engineering, Germany). This device uses a dual-beam SD-OCT and a confocal laser scanning ophthalmoscope that works by emitting a superluminescent diode light with a center wavelength of 870 nm and an infrared scan to simultaneously provide images of ocular microstructures. For RNFL measurements 16 consecutive circular B-scans are automatically averaged and compared with a normative database. The signal of the examination had to be at least 15 dB to be included in the study; a commonly used cut off level for obtained scans[29,30]. The eye with the lower global RNFL thickness values for a given patient was considered to be the worse eye with respect to structural damage on SD-OCT testing.

### Perimetry

Standard automated perimetry is currently the gold standard for visual field testing. Visual field was assessed using the Humphrey Field Analyzer (HFA, Humphrey Instruments Inc., CA, USA) and the SITA 30-2 standard algorithm; whereby the parameters mean deviation (MD) and the pattern standard deviation (PSD) were recorded. A reliable visual field test was defined as having a <25% rate of fixation losses, and <20% false positive and false negative values. The MD was used to determine, which eye of each patient was the better or worse eye; the eye with the lower MD was considered to be the worse eye (more glaucomatous damage). Glaucoma staging was determined following a simplified Hodapp-Anderson-Parrish modification [31,32] as early (=>-6dB), moderate (>-6dB and =>-12dB) and severe (

<-12dB) glaucoma

. 

### Statistical analysis

We performed Rasch analysis on the German GAL-9 using Winsteps software (version 3.68), Chicago, Illinois, USA. The Andrich rating scale model was used and four rating scales were applied (see [19] [26] for more details).

Visual acuity mean values were calculated using log minimum angle of resolution (logMAR) values. Descriptive statistical analyses were performed to characterize the patients’ clinical, functional and structural data. Difference testing between eyes was done using the Student t-test. For correlation analysis, the Pearson correlation coefficient was calculated for normally distributed values (according to the Kolmogorov-Smirnov test), otherwise the Spearman’s rank coefficient was used. Relationships were also tested with multiple linear regression; t-based 95% confidence intervals (CIs) for the regression coefficients were used. All statistical analyses were performed with the SPSS statistical software (Version 17.0, SPSS Science, Chicago, IL).

## Results

A total of 88 patients were included (176 eyes); fifty-one (58.0%) patients were female. Perimetry, GAL-9 testing and SD-OCT of the peripapillary RNFL were obtained from all patients. The best-corrected VA of the MD-guided better eye was 20/28 (logMAR 0.14) and of the worse eye 20/33 (logMAR 0.21, Table 1). Central corneal thickness and IOP were not significantly different in MD-guided better and worse eyes on Student-t testing. On the basis of MD of the better eyes, 72.7%, 15.9% and 11.4% of the study population were classified as having early, moderate and advanced form of glaucoma following the modified grading system of Hodapp-Anderson-Parrish. When considering worse eyes, 50%, 26.1% and 23.9% had early, moderate and advanced glaucoma. 

**Table 1:** Demographic, biometric and clinical characteristics including the spectral-domain optical coherence tomography (SD-OCT) assessed retinal nerve fibre layer (RNFL) thickness (mean values +/- 1 SD).

Age (years)		63.2 +/- 13.4	
GAL-9 person estimate		-2.45 +/- 2.01	
		MD guided b/e	MD guided w/e
VA, logMAR		0.14 +/- 0.23	0.21 +/- 0.28
IOP (mmHg)		13.7 +/- 3.23	13.7 +/- 4.19
CCT (µm)		548 +/- 35	548 +/- 36
Mean defect (dB)		-4.0 +/- 5.1	-7.1 +/- 5.9
Pattern standard deviation		4.2 +/- 3.0	6.1 +/- 3.9
SD-OCT sector (µm)			
	*temporal*	64 +/- 14	55 +/- 19
	*temporal-superior*	95 +/- 30	85 +/- 28
	*nasal-superior*	72 +/- 18	70 +/- 22
	*nasal*	60 +/- 20	60 +/- 16
	*nasal-inferior*	86 +/- 22	77 +/- 21
	*temporal-inferior*	101 +/- 38	85 +/- 38

Mean RNFL thickness was lower in each sector in the MD-guided worse eyes (Table 1). Rasch analysis revealed GAL-9 values from -6.13 (best glaucoma-specific visual function) to 2.67 (worst glaucoma-specific visual function).

### Correlation analyses

RNFL thickness for all sectors and GAL-9 person estimates were normally distributed on Kolmogorov-Smirnov testing, VA and perimetrical data were not normally distributed. The most relevant correlations are displayed in Table 2. There were no significant correlations between the RNFL thickness of the better eyes and GAL-9 assessed functional impairment. However, there were moderate significant correlations between the temporal-superior and temporal-inferior sector of the worse eye and GAL-9 scores (r=-0.298, p=0.011 and r=-0.251, p=0.033, respectively). The correlations are negative, because patients with better functioning are located on the negative side of the logit scale.

**Table 2:** Correlation analysis between important functional parameters and IOP, CCT and RNFL sectors (T: temporal, TS: temporal superior, NS: nasal superior, N: nasal, NI: nasal inferior, TI: temporal inferior, G: global) for better eye (b/e) and worse eye (w/e), respectively.

	GAL-9		VA b/e		VA w/e		MD b/e		MD w/e	
	correl.	p	correl.	p	correl.	p	correl.	p	correl.	p
Age	*0.102*	*0.398*	**0.287**	**0.021**	**0.356**	**0.004**	-0.148	0.245	-0.222	0.088
VA b/e	**0.303**	**0.014**					**-0.330**	**0.010**	-0.070	0.594
VA w/e	**0.416**	**0.000**					**-0.335**	**0.010**	**-0.316**	**0.018**
MD b/e	**-0.479**	**0.000**								
MD w/e	**-0.373**	**0.000**								
IOP b/e	*-0.019*	*0.816*	-0.144	0.087	-0.092	0.277	0.107	0.417	-0.004	0.976
IOP w/e	*-0.093*	*0.268*	0.054	0.524	-0.162	0.053	-0.024	0.857	-0.171	0.203
CCT b/e	*0.136*	*0.134*	0.072	0.432	-0.003	0.973	-0.063	0.522	0.149	0.153
CCT w/e	*0.048*	*0.605*	0.093	0.317	0.038	0.680	-0.071	0.473	0.141	0.175
RNFL T b/e	*0.113*	*0.339*	-0.041	0.742	0.034	0.786	**0.265**	**0.033**	0.154	0.231
RNFL T w/e	*0.016*	*0.895*	0.073	0.558	**-0.328**	**0.007**	**0.314**	**0.012**	**0.436**	**0.000**
RNFL TS b/e	*-0.081*	*0.498*	-0.129	0.298	0.037	0.764	**0.456**	**0.000**	**0.311**	**0.013**
RNFL TS w/e	***-0.298***	***0.011***	**-0.247**	**0.045**	**-0.247**	**0.046**	**0.433**	**0.000**	**0.527**	**0.000**
RNFL NS b/e	*-0.021*	*0.861*	-0.001	0.990	-0.058	0.642	**0.287**	**0.020**	0.158	0.220
RNFL NS w/e	*0.030*	*0.804*	-0.073	0.562	0.032	0.798	0.146	0.250	**0.263**	**0.041**
RNFL N b/e	*-0.106*	*0.370*	0.044	0.721	-0.173	0.162	**0.303**	**0.014**	0.193	0.133
RNFL N w/e	*0.118*	*0.325*	0.012	0.922	-0.108	0.388	0.215	0.088	**0.372**	**0.003**
RNFL NI b/e	*-0.197*	*0.094*	-0.104	0.403	-0.178	0.149	**0.520**	**0.000**	**0.331**	**0.009**
RNFL NI w/e	*-0.059*	*0.620*	0.049	0.697	**-0.317**	**0.009**	**0.256**	**0.046**	**0.399**	**0.001**
RNFL TI b/e	*-0.221*	*0.061*	-0.232	0.059	-0.219	0.075	**0.639**	**0.000**	**0.562**	**0.000**
RNFL TI w/e	***-0.251***	***0.033***	0.084	0.502	**-0.320**	**0.009**	**0.394**	**0.001**	**0.671**	**0.000**
RNFL G b/e	*-0.174*	*-0.14*	-0.141	0.254	-0.112	0.336	**0.673**	**0.000**	**0.514**	**0.000**
RNFL G w/e	*-0.075*	*0.530*	-0.010	0.937	**-0.318**	**0.009**	**0.435**	**0.000**	**0.668**	**0.000**

Cursive numbers are Pearson´s correlation coefficients, otherwise Spearman rank coefficients are displayed. Significant coefficients (p<0.05) are bold.

The MD values of better and worse eyes were highly correlated with the RNFL thickness measurements, and correlations between RNFL thickness and MD were higher for the worse eye. The highest correlations of all RNFL sectors were observed for the temporal-superior and temporal-inferior sector of the worse eye with the MD of the worse eye (r=0.527, p<0.001 and r=0.671, p<0.001, respectively). The global RNFL of the worse eye was highly correlated with the MD of the worse eye (r=0.668, p<0.001). 

Regarding functional parameters and patient-reported visual functioning, there was a trend to a higher correlation of GAL-9 scores with the MD of the better eye than GAL-9 score with the MD of the worse eye (r=-0.479, p<0.001 and r=-0.373, p<0.001).

### Linear Regression Models

Linear regression analyses were performed to analyze the impact of RNFL loss on functional parameters and patient-reported visual functioning. SD-OCT-assessed RNFL thickness of each sector of the MD-guided better or worse eye entered linear regression to predict GAL-9 person estimates (Table 3). 

**Table 3:** Univariate linear regression.

		Adj. R^2^	Coefficient	P		Adj. R^2^	Coefficient	p
Age		0.032	0.179	0.023				
VA	b/e	**0.086**	**0.294**	**0.000**	w/e	**0.196**	**0.443**	**0.000**
IOP	b/e	0.001	-0.019	0.816	w/e	0.009	-0.093	0.268
CCT	b/e	0.019	0.136	0.134	w/e	0.002	0.048	0.605
MD	b/e	**0.279**	**-0.528**	**0.000**	w/e	**0.196**	**-0.443**	**0.000**
PSD	b/e	**0.144**	**0.379**	**0.000**	w/e	0.021	0.143	0.112
CDR	b/e	0.047	-0.217	0.095	w/e	0.020	-0.142	0.280
RNFL T	b/e	0.013	0.113	0.339	w/e	0.001	0.016	0.895
RNFL TS	b/e	0.007	-0.081	0.498	w/e	**0.089**	**-0.298**	**0.011**
RNFL NS	b/e	0.001	-0.021	0.861	w/e	0.001	0.030	0.804
RNFL N	b/e	0.011	-0.106	0.370	w/e	0.014	0.118	0.325
RNFL NI	b/e	0.039	-0.197	0.094	w/e	0.004	-0.059	0.620
RNFL TI	b/e	0.049	-0.221	0.061	w/e	0.063	-0.251	0.033
RNFL G	b/e	0.030	-0.174	0.141	w/e	0.006	-0.075	0.530

Dependent variable GAL-9. Visual acuity (VA) and mean defect (MD) were significant predictors for GAL-9 scores with the MD of the better eye revealing best modelling (R^2^=0.279). The only significant predictor of structural parameters was the retinal nerve fibre layer (RNFL) of the temporal superior (TS) sector of the worse eye. IOP: intraocular pressure, CCT: central corneal thickness, PSD: pattern standard deviation, CDR: cup-to-disc ratio.

A multivariate linear regression analysis was performed to predict GAL-9 person estimates entering significant structural and functional parameters. The best model to predict GAL-9 person estimates includes the VA of the worse eye and the MD of the better explaining 33.4% of the variance in patient-reported visual functioning (Table 4). This represents an improvement over VA w/e 19.6% and MD b/e 27.9% alone. Adding SD-OCT assessed RNFL metrics did not enhance the predictive power in multivariate linear regression and are therefore not presented.

**Table 4:** Best model in multiple regression analysis to predict GAL-9 scores.

	Adjusted R^2^	Coefficient	p
GAL-9	0.334		
VA of w/e		0.309	0.014
MD of b/e		-0.373	0.003

Adding any sector of the RNFL or age did not enhance the predictive power.

## Discussion

To the best of the authors´ knowledge, this is the first study that compares glaucomatous RNFL thickness and patient-reported visual functioning. It could be hypothesized that structural change should lead to visual change, which in turn should impact person-reported measurements. However, a major finding of our study is that the RNFL is not well correlated or highly predictive for the GAL-9. There seems to be a degree of separation between structural and patient-reported changes. The functional parameters visual acuity and MD predict GAL-9 scores much better than the SD-OCT assessed structural parameter RNFL. 

In studies that explore structure-function relationships in ophthalmology and in glaucoma in particular, it has to be considered that most patients have a worse and a better eye[33]. The overall best model to predict GAL-9 scores include VA of the worse eye and perimetric MD of the better eye (R^2^=0.334). The finding that the VA of the worse eye is a better predictor for functional impairment is noteworthy, as numerous previous publications suggested that patient-reported visual functioning is at least partially driven by the eye with the better visual acuity [34,35]; particularly in eyes with cataract. Therefore, this is an intriguing finding, although this has been found in glaucoma before. Our findings are consistent with a recent study by Gothwal et al., who report a far better correlation of the patient-reported visual functioning using the Glaucoma Activity Limitation 10 score with the VA of the worse eye (r=0.49) than with the VA of the better eye (r=0.35) [36]. Gothwal et al hypothesized that this may be due to the limited variance in the good eye reducing the power to detect a relationship. However, in our data this can only be a minor role with the better eye and the worse eye having similar variance (SD b/e ±0.23, w/e ±0.28). The finding of a stronger influence of the MD of the better eye on patient-reported visual functioning has also been reported previously [36,37]. Other studies in glaucoma have reported that the worse eye is the one that correlates better with function, when function was directly measured and set in comparison to clinical values, including visual fields[38-40]. This suggests that vision loss in one eye in glaucoma has an effect on the patient that cannot be overcome by the better eye. It has been observed in other eye diseases that the worse eye with stable function of the fellow eye has significant impact on patients´ reported outcome [34,41]. Bressler et al. reported a clinically significant increase of patients´ quality of life when the worse eye with wet age-related macular degeneration was treated, although the binocular visual acuity did not increase[42]. 

As expected, structural parameters and perimetric performance were highly correlated, with a correlation coefficient of 0.668 between the global RNFL of the worse eye was and the MD of the worse eye. This is because the measured structural changes directly related to the loss of visual field. This compares favorably with previous studies[3]. [4] However, this global impairment of the RNFL does not directly result in a global reduction in patient-reported visual functioning. This is likely due to the role of the fellow eye in driving patient-reported visual functioning, the importance of RNFL loss for patient-reported visual functioning and patient adaptation to these losses. Although correlations of structural parameters with GAL-9 scores were rather low, structural characteristics displayed by RNFL thickness of the eye with the worse glaucomatous damage is better correlated with GAL-9 scores than the better eye. It can be suggested that losses in the worse eye are not fully compensated by the better eye resulting in functional losses for the person. The significant correlation with VA (r=-0.320, p=0.009) in the worse eye for the temporal-inferior peripapillary sector could be explained by the fact, that this sector receives the majority of the nerve fibers from the papillo-macular bundle given the off-center position of the optic nerve head; these fibers carry visual acuity signal. One previous study has shown that inferio-temporal field loss is the most predictive of self-reported reading and other visual functioning[43]. Another explanation for why worse eyes´ structural changes may have driven GAL-9 outcomes more than the better eye could be a distribution of visual field damage including many subjects with unilateral visual field damage and near-normal better eye function, but this was not the case in our sample. By selecting patients with rather good visual acuity some bias could have been added to the results.

For patients with good binocular visual acuity, the worse eye may play a significant role in their performance, whereas for patients with poor binocular visual acuity (e.g. 20/100 to 20/200), the better eye may be more important. In this study, there is more variance in the worse eye data which improves the probability of a strong correlation. However, most patients with a diagnosis of glaucoma have good central visual acuity, and therefore, the results appear to be representative of a randomly selected glaucoma population. 

A strength of this study is the use of Rasch-scaled person estimates of the GAL-9. The GAL-9 was shown to have excellent psychometric characteristics except for targeting of item difficulty to person ability[19]. Poor targeting is an inevitable attribute as most of the glaucoma patients have little difficulty in performing everyday tasks[44,45]. Furthermore, the GAL-9 does not reflect overall quality of life as important aspects like anxiety or depression are not adequately addressed [46]. Better questionnaires in the future might enable measurement beyond visual functioning to include overall quality of life, which might fit better in models including structural changes. Item banking approaches for glaucoma quality of life measurement are under development and these may provide better options for research in this field[47,48]. It must be considered that we did not add a comorbidity index, which could be a potential confounder. Another possible confounder could be that trying to find early glaucomatous changes with multiple diagnostic tools could make the patients worry unnecessarily and affect their quality of life. However, the GAL-9 focusses on activity limitation rather than worry, therefore this possible confounder is minimized. One should also keep in mind that quality of life and activity limitation is partially dependent on patients´ age. We did not adjust our data for age as there is not sufficient data at the moment to perform this standardization. 

The ultimate gain of glaucoma therapy is to prevent loss of patients´ quality of life. High quality measurement of structural, visual and patient-reported outcomes and an understanding of their inter-relationships is critical to achieving this goal. It would be of interest to investigate longitudinally, whether localized RNFL thinning or other structural change in glaucoma can predict future deficits in patient-reported visual functioning. New measurements of structural changes, e.g. the optic nerve head using the minimum rim width by SD-OCT [49], might reveal higher correlations with visual functioning and might support the theory that the worse eye has a higher impact in glaucoma than the better eye.

## Conclusions

Structural measurements of the peripapillary RNFL thickness by SD-OCT are weak predictive factors of patient-reported visual functioning as assessed by the GAL-9. The best predictors of patient-reported visual functioning are the visual acuity of the worse eye and the MD of the better eye.
